# External validation of a prognostic index

**DOI:** 10.1186/1745-6215-14-S1-P43

**Published:** 2013-11-29

**Authors:** Laura Bonnett, Anthony Marson, Tony Johnson, Lois Kim, Ley Sander, Nicholas Lawn, Ettore Beghi, Maurizio Leone, Catrin Tudur Smith

**Affiliations:** 1 University of Liverpool, Liverpool, UK; 2 MRC Biostatistics Unit, Cambridge, UK; 3 MRC Clinical Trials Unit, London, UK; 4 London School of Hygiene & Tropical Medicine, London, UK; 5 Clinica Neurologica, Ospedale Maggiore della Carita, Novara, Italy; 6 IRCCS Istituto di Ricerche Farmacologiche Mario Negri, Milano, Italy; 7 Royal Perth and Fremantle Hospitals, Perth, Australia; 8 UCL, London, UK; 9 Epilepsy Society, Chalfont St Peter, UK

## Background

Prognostic indices, derived from prognostic models, can be used to stratify patients in clinical trials. This includes stratifying eligible patients for randomisation, stratifying randomisation, or identifying subgroups for the trial analysis. However, before an index can be used for these purposes, it needs to be validated externally in independent data. We demonstrate methods of external validation, including methods for handling a covariate missing from the validation dataset, via a prognostic model for risk of seizure recurrence following a first ever seizure.

## Methods

Three independent datasets were obtained. External validation was evaluated for each dataset via discrimination using Harrell's c-index. Calibration plots were also considered. Five imputation methods were examined to handle a covariate missing from one validation dataset. These included hot deck imputation and multiple imputation.

## Results

Trial data for 620 people with epilepsy was used to develop the original model; the validation datasets consisted of 274, 307 and 847 trial participants respectively. The model generalised relatively well to the other datasets - two out of three calibration plots demonstrated excellent fits while two of the three datasets had c-indexes within 0.01 of each other. All five methods of imputation performed fairly similarly.

## Conclusions

Prognostic models, and consequently prognostic indices, can be validated by considering calibration and discrimination methods. Although there are limitations to external validation, it is still a necessary part of modelling.

